# Adaptive Strategies and Person-Environment Fit among Functionally Limited Older Adults Aging in Place: A Mixed Methods Approach

**DOI:** 10.3390/ijerph120911954

**Published:** 2015-09-23

**Authors:** Laura L. Lien, Carmen D. Steggell, Susanne Iwarsson

**Affiliations:** 1 School of Design and Human Environment, College of Business, Oregon State University, 228 Milam Hall, Corvallis, OR 97331, USA; E-Mail: carmen.steggell@gmail.com; 2 Department of Rehabilitation Science, School of Public Health and Health Professions, University at Buffalo, SUNY, 501 Kimball Tower, Buffalo, NY 14214, USA; 3 Department of Health Sciences, Faculty of Medicine, Lund University, Sweden, Box 157, 221-00 Lund, Sweden; E-Mail: Susanne.Iwarsson@med.lu.se

**Keywords:** accessibility, usability, person-environment fit, adaptive behaviors

## Abstract

Older adults prefer to age in place, necessitating a match between person and environment, or person-environment (P-E) fit. In occupational therapy practice, home modifications can support independence, but more knowledge is needed to optimize interventions targeting the housing situation of older adults. In response, this study aimed to explore the accessibility and usability of the home environment to further understand adaptive environmental behaviors. Mixed methods data were collected using objective and perceived indicators of P-E fit among 12 older adults living in community-dwelling housing. Quantitative data described objective P-E fit in terms of accessibility, while qualitative data explored perceived P-E fit in terms of usability. While accessibility problems were prevalent, participants’ perceptions of usability revealed a range of adaptive environmental behaviors employed to meet functional needs. A closer examination of the P-E interaction suggests that objective accessibility does not always stipulate perceived usability, which appears to be malleable with age, self-perception, and functional competency. Findings stress the importance of evaluating both objective and perceived indicators of P-E fit to provide housing interventions that support independence. Further exploration of adaptive processes in older age may serve to deepen our understanding of both P-E fit frameworks and theoretical models of aging well.

## 1. Introduction

The ability to remain independent well into old age is a fundamental desire among older adults. In fact, the majority of older adults worldwide live in community-dwelling housing (*i.e.*, independent living environments) *vs.* supportive housing facilities [1]. Declines in health and physical environmental barriers within the home, however, can challenge older adults’ abilities to remain at home [2,3]. In occupational therapy practice, individual home modifications are often used to support aging in place [4], but more knowledge is needed to develop and optimize interventions targeting the housing environment of older adults.

Within our global aging society, the proportion of older adults over the age of 60 is steadily growing, changing the dynamics of living arrangements and housing provision in drastic ways [5]. According to the United States (U.S.) Census Bureau [6], the U.S. population aged 65 and older is expected to rise by close to 76% by the year 2050, and similar developments are seen in other continents and countries. Increases in disability rates [7] and changes in the demographics of the labor force, community services, and health programs may threaten the ability of older adults to live independently and age in place [2]. Appropriate housing in older age not only provides residential stability and personal meaning, but also environmental support to overcome or compensate for declines in functional capacity common with age [8].

According to Lawton and Nahemow’s ecological theory of aging (ETA) [9], the performance of and comfort with daily necessary and desired activities is possible when an appropriate match between a person and his/her environment is achieved. This match, or zone of maximum performance and comfort, is known as person-environment (P-E) fit. P-E fit is both objective and perceived, and is important in understanding the dynamic relationship between people and their environments, especially in older age [10]. The interaction between a person and his/her environment describes how older adults adapt themselves or their environments to achieve a match between competence and environmental press [11]. The specific adaptive environmental behaviors employed by older adults to meet actual or perceived functional needs, however, are little understood both conceptually and empirically [12].

To deepen our understanding of adaptation to P-E fit challenges, Baltes and Baltes’ [13] selection, optimization, and compensation (SOC) model might be helpful. This model suggests that physical, social, and psychological functional losses in old age can be mitigated through three types of adaptive behaviors: *selection* (choice of desired outcomes or goals), *optimization* (skills or strategies to achieve goal-related success), and *compensation* (the maintenance of desired functional outcomes in response to losses in goal-related achievement) [14]. These processes can be intentional (and therefore conscious) or unintentional (subconscious), and internally or externally motivated. Like the P-E fit framework, the SOC model focuses on both objective and perceived aspects of person and context, or surrounding environmental conditions [13]. In contrast to the P-E fit framework, however, the SOC model emphases specific behaviors in response to functional loss and/or environmental challenges that help people adapt to their changing self and environment over the lifespan.

Home modifications and provision of assistive technologies are common interventions that support independent living among older adults [4,15]. Modifications are defined as adaptations to the physical environment, ranging from the simple elimination of slip and trip hazards (e.g., throw rugs) or the installation of grab bars or railings to complex remodeling of living spaces to accommodate daily personal (P-) and instrumental activities of daily living (I-ADL) [15]. Independent living in older age is also supported with assistive technologies and devices, such as hearing aids, screen readers, and personal equipment (e.g., walkers, canes) [15,16]. There are some programs in the U.S. that assist older adults in identifying and obtaining modifications and assistive technologies; however, budget limitations, instability in funding sources, and uneven coverage in many geographic locations results in many older adults paying for such interventions out of pocket [15].

While studies on the effects of such interventions have been published [17,18], few studies have explored the specific adaptive environmental behaviors utilized by older adults to overcome or compensate for a lack of P-E fit [12]. In addition to the need for research that supports the theoretical development in this area of inquiry, a better understanding of how people interrelate with their environments can assist in the creation of appropriate policy and practice interventions to support independence in older age. In response, this study aimed to examine objective measures of accessibility in concordance with perceived usability of the home environment among older adults with functional limitations. Using the ETA [9] and SOC model [13] as theoretical frameworks, a better understanding of the adaptive environmental behaviors employed to achieve P-E fit in the home in older age is possible.

## 2. Design and Methods

A mixed methods embedded approach was used, which “nests,” or embeds, one method (*i.e.*, quantitative) within another (*i.e.*, qualitative). Such designs use a secondary but supportive form of data to inform the primary research method guiding the project [19]. In this study, quantitative data were collected using objective measures of P-E fit (*i.e.*, accessibility) among functionally limited older adult participants in community-dwelling housing in the U.S. Qualitative interviews focused on participants’ perceived P-E fit (*i.e.*, the usability of their home), using results from objective assessments of accessibility as a prompt. The ETA [9] provided the theoretical framework essential for the incorporation of P-E fit indicators, and the SOC model [13] was used to inform our understanding of the adaptive environmental behaviors participants employed in response to P-E fit challenges.

### 2.1. Study District

Participants lived within the city center through the more rural surrounding areas of a smaller urban region of the Pacific Northwest in the U.S. According to the American Community Survey [20], this region had a population <100,000, and 13.8% were aged 65 and older. Almost 90% of the population was white. Mean annual income was $65,000, with 31.6% of the population earning $75,000 or more. Upwards of 52.2% of the population had a Bachelor’s degree or higher, which is likely attributable to its proximity to a major research university. In 2013, 57% of housing units were owner-occupied. Generally, this region is highly educated, affluent, and predominantly white.

### 2.2. Sample

To recruit initial participants, comprehensive e-mail announcements were sent to all adults aged 65 and older living within the study region that were members of a research center’s participant registry database of older adults willing to or who already regularly participated in research studies at the university (N = 50). This age range was chosen to comply with the U.S. standard classification of older adulthood, as well as follow the traditional approach used in gerontological research. Recruitment e-mails included a list of inclusion criteria requiring participants to be aged 65 years or older, living independently in community-dwelling housing (*i.e.*, independent living environments without formal support), and able to identify with one or more of the functional limitations assessed by the Housing Enabler (HE) [21].

Initial participants recruited through registry e-mails (N = 5) were scheduled for home visits and later helped identify additional participants (N = 7) with varying types of functional limitations and home environments within the study region. This recruitment continued until a diverse group of participants (with respect to type of functional limitations and home environment) was ascertained and data saturation was achieved. This approach offered a range of different perspectives through which accessibility, usability, and adaptive environmental behaviors among functionally limited older adults living independently could be explored.

The final study sample consisted of 12 participants, 66–89 years of age (characteristics summarized in Table 1). One participant had only one functional limitation; the other 11 each had 2–7 limitations. The most common functional limitations involved mobility restrictions—more specifically, poor balance, incoordination, and limitations of stamina. Functional limitations in upper and lower extremity function characterized the next most common type, primarily related to hip, knee, shoulder, and wrist injuries or arthritis. Five of the 12 participants used a mobility device, ranging from the use of a cane in the exterior environment to dependence on a wheelchair both indoors and outdoors. All participants utilized at least one assistive device, and all but one had made some type of home modification after establishing residence.

The majority of participants lived in single-family, detached, owner-occupied homes (see Table 1). Time of residence ranged from one month to 47 years, with five participants having lived in their homes for over 40 years. Three participants lived in single-family houses either custom designed to accommodate aging or selected to support potential age-related needs. Two participants lived in independent age-restricted housing on a Continuing Care Retirement Community (CCRC) campus, and were the only multifamily housing residents.

**Table 1:** Demographic Characteristics of Study Participants, N = 12.

Characteristic	N
Sex	
Men	5
Women	7
Age	
65–69	1
70–79	6
80–89	5
Type of Functional Limitation	
Visual	4
Hearing	7
Mobility Restrictions	10
Upper extremity	8
Lower extremity	8
Mobility device	5
Type of Housing	
Single-family, detached	7
Age-specific	2
Custom/planned for age	3
Years Lived in Current Home	
0–10	4
11–20	3
21+	5

Notes: Functional limitation items were grouped together by type for the purposes of demographic characteristics. Visual = vision restrictions in one or both eyes, blindness. Hearing = hearing restrictions in one or both ears, loss of hearing, hearing aids. Mobility Restrictions = poor balance, incoordination, limitations of stamina, difficulty in moving head. Upper extremity = reduced arm/hand function in one or both arms and hands, reduced fine motor skills, loss of function. Lower extremity = reduced mobility/strength in spine or joints of one or both legs. Mobility device = whole or partial dependence on walking aids (canes, crutches, sticks, walkers, *etc.*) and/or wheelchair. Adapted from *Housing Enabler: A Method for Rating/Screening and Analyzing Accessibility Problems in Housing* [21].

### 2.3. Data Collection and Procedures

Two scheduled visits per participant were completed for the data collection. The first author completed visits for each participant 1-1½ weeks apart. Each participant visit ranged anywhere from 45 min to 3½ hours, depending on the size of the home and the in-depth discussion of interview questions. Most accessibility assessments were completed within 1 h, while the perceptions of usability interviews often took 1½-2 h.

#### 2.3.1. Quantitative Data

Quantitative data were collected during the first visit. Demographic information for each participant was recorded, including sex, age, type of home, and years lived in present dwelling. Objective P-E fit data were gathered using a U.S. version [22] of the Housing Enabler (HE) [21]. The HE, originally developed in Sweden, provides an objective measure of P-E fit through a three-step assessment process: (1) an evaluation of a person’s functional limitations (12 items; ranging from limitations in hearing or vision to reduction or loss in upper and lower extremity function, see Table 1) and dependence on mobility devices (two items, present/not present; included here as both a consequence of severe limitations in function and an extension of the person which together interacts with the built environment); (2) the identification of environmental barriers in the home and exterior surroundings (161 items, present/not present); and (3) calculation of the resultant accessibility score. This score is based on a per item 0–4 predefined scoring system based on the relationship between a person and his/her home environment that produces a total accessibility score ranging from 0 to 1832, with higher scores denoting more accessibility problems. Based on the scoring system, also rank-ordered environmental barriers are computed [21].

Environmental barrier items within the HE assessment are defined based on national accessibility standards and guidelines for housing design; therefore, the primary focus of the instrument is on accessibility problems generated by the encounter of an individual with physical, sensory, and cognitive functional limitations and the built environment. As the HE instrument originated in Sweden, valid use of the instrument in other countries requires careful adaptation to ensure strict adherence to country-specific accessibility standards for housing design [23]. The U.S. version of the HE instrument [22] was adapted for use through collaboration with its originators to provide a reliable and valid method of determining accessibility problems within U.S. home environments based on associated accessibility standards. It was used in this study to assess and present the magnitude of accessibility problems and weighted environmental barriers within participants’ home environments during the qualitative phase of data collection to elicit perceptions of accessibility and usability in the home, as well as to incorporate such data into the mixed methods analysis.

Reliable and valid use of the HE tool has been shown across European contexts. Previous inter-rater agreement findings have shown moderate to good agreement (the original Swedish HE instrument: κ = 0.68, and the cross-national adaptation for the ENABLE-AGE project: κ = 0.50 with 85% agreement) [24]. Efficient and consistent use of the tool requires specialized training and practice [21]. The first author was formally trained by completing a five-day HE course given by the originator of the instrument (last author). This training, along with her principal involvement in the adaptation process of the HE to U.S. applications [22], provided the necessary credentials to utilize the U.S. version of the HE in the present study.

#### 2.3.2. Qualitative Data

**Figure 1:**
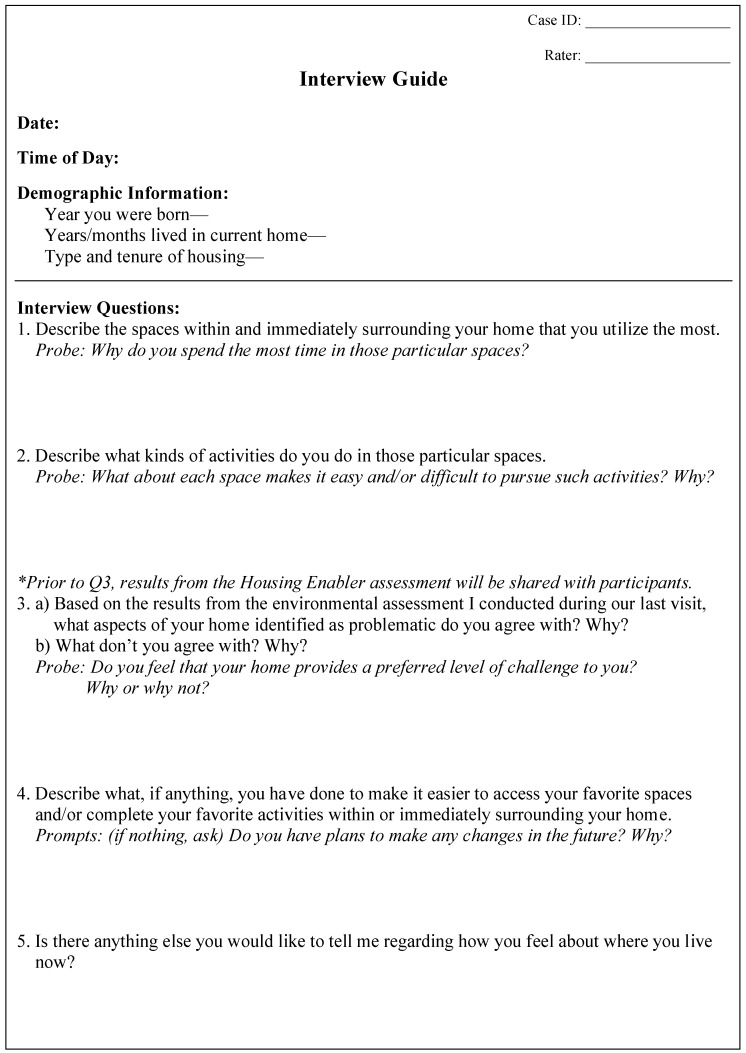
Semi-structured interview guide using the SOC model [13] as a framework.

**Figure 2:**
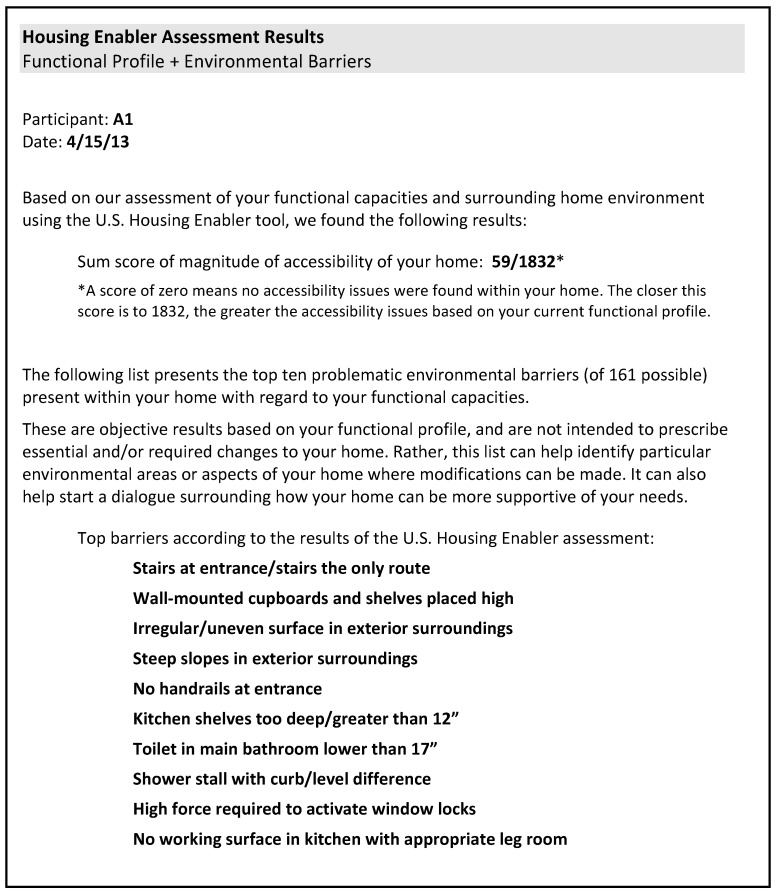
Example of HE report sheet presented to participants during qualitative interviews. Report described magnitude of accessibility problems score and top ten ranked environmental barriers present within their home environment.

The primary form of qualitative data collected was from semi- structured interviews conducted during the second visit. Using the SOC model as a framework [13] (see Figure 1), five questions aimed at describing adaptive strategies with respect to the *selection* of daily activities, the *optimization* of environmental spaces, and the *compensation* for losses in functional capacity and consequent need for personal and environmental support were asked of each participant. Quantitative P-E fit results stemming from the individual U.S. HE assessments were shared with each participant in the same manner via a study-specific report sheet presented during the interview prior to Question Three (see Figure 1). The report described the accessibility problems score as well as the top ten rank-ordered environmental barriers identified within each individual dwelling (see Figure 2).

Sharing the HE results with participants during the qualitative interview helped solicit perspectives and experiences of accessibility (that is, objective P-E fit) problems within the home environment with respect to passive or proactive approaches to daily activity as suggested by the ecological theory of aging (ETA) [9]. Furthermore, this process assisted in highlighting the adaptive strategies that participants employed to achieve or modify daily activities in response to functional loss and/or environmental challenge through selection, optimization, or compensation behaviors. In other words, incorporating the objective HE data within the interview intended to shed light on adaptation behaviors used to achieve P-E fit. To further encourage participant discussion, probing questions were asked throughout, and were tailored to elicit each participant’s personal and unique lived experience through his/her own words. All interviews were audio taped for accuracy in transcription.

Direct observations, field notes, and photographs taken during both visits further supported the interview data. This approach is valuable when exploring behaviors or the lived experience of participants, as it helps contextualize participant responses [25]. While the interviews pointedly asked about the activities participants regularly performed at home, observations of activity performance and reflective field notes provided additional validity. Photographs of participants’ physical home environments were taken during the administration of the HE assessment at the first visit, but were occasionally supplemented in subsequent visits, depending on the time of day or weather. Photographs visually described how the participants used, performed, and engaged in activities within their home, and also helped corroborate or juxtapose participant interview responses by assuming an objective role contrasting or supporting participants’ perceptions of P-E fit.

### 2.4. Data Analysis

Objective accessibility (as measured in quantitative assessments) and perceived usability (as captured through semi-structured interviews) provided the context for the analysis. Descriptive statistics were used to analyze the quantitative data. The magnitude of accessibility problems and type and prevalence of top-ten ranked environmental barriers assessed through the U.S. version [22] of the HE [21] described the objective P-E fit of the participants (see Table 2 and Table 3). Integrated in the analysis process, the quantitative data from the HE assessment was used to inform the primarily qualitative data as recommended in embedded mixed methods approaches [19]. Data analysis was primarily inductive in its consideration of the qualitative data as a means to generate themes related to adaptive P-E interactions with respect to the two theoretical models guiding the study.

**Table 2:** Objective Indicators of Person-Environment Fit in the Home Environments of Study Participants, N = 12.

Participant ID	Age	Functional Limitations ^a^	Mobility Devices, n ^a^	Assistive Devices, n ^b^	Mods ^b^	Magnitude of Accessibility Problems ^c^
A1	77	4	None	8	1	59
A2	70	4	None	2	3	218
A3	86	4	None	6	3	155
A4	78	1	None	2	4	22
A5	89	7	2	3	2	251
A6	79	4	1	3	4	249
A7	87	2	None	2	4	114
A8	77	4	2	3	None	89
A9	73	4	2	1	3	233
A10	88	6	2	2	1	170
A11	66	3	None	1	1	79
A12	80	4	None	4	4	108

Notes: **^a^** U.S. Housing Enabler [22] assessment, number of functional limitations (12 items), number of mobility devices (2 items); **^b^** Number of assistive devices and home modifications (Mods) based on participant observation and interview responses; **^c^** Magnitude of accessibility problems calculated through the full U.S. Housing Enabler [22]. Sample-specific scores range from zero to 400, with zero indicating no accessibility problems and 400 indicating more severe accessibility problems within the home environment. The theoretical maximum score is 1832 [21].

**Table 3:** Group-Based Analysis of Prevalence of Top Ten Ranked Environmental Barriers (Displayed in Ranked Order) in the Home Environments of Study Participants, N = 12.

Rank	U.S. HE Item (Ranked Environmental Barrier)	Prevalence, N Cases
1	Wall-mounted cupboards and shelves placed high in kitchen & laundry	10
2	Stairs the only route at entrance	9
3	Kitchen cupboard shelves too deep	9
4	No grab bars at shower/bath and/or toilet	7
5	High thresholds and/or steps at entrance	11
6	High thresholds/level difference/step to sitting out space	11
7	Garbage bin can only be reached via steps or other difference in level	10
8	Exterior routes with steps	8
9	No handrails/handrails on one side only on stairs at entrance	9
10	Controls in high/inaccessible position in kitchen & laundry	12

Notes: Ranked environmental barriers identified through use of the U.S. HE instrument [22].

The qualitative data were analyzed using a content analysis approach to generate themes [26]. In this inductive process, we analyzed and synthesized the findings using a template approach [27] that derived codes based on the research questions and theoretical categories (*i.e.*, P-E fit and adaptive behaviors). Significant statements were identified and considered with respect to overall meaning and thereafter categorized into themes that were then compared back to the theoretical categories. This process was fluid and flexible, resulting in final themes that were guided by the theoretical frameworks.

To achieve this, multiple steps were performed. First, the audiotapes from each interview were listened to by the first author multiple times to gain familiarity with the interview data and the settings that contextualized the data collection. All interviews were transcribed verbatim by the first author, and were formatted in one document for the purpose of exploring themes. The first two authors individually and separately read the transcriptions multiple times to identify significant statements that encapsulated the phenomenon of adaptive behaviors employed to achieve P-E fit in the home environment. Emerging themes were then cross-analyzed with observations, field notes, photographs, and the quantitative results to further validate findings. Trustworthiness was established through multiple discussions between the first and second author of evolving themes until consensus was found. Validity of themes was strengthened by the identification of significant quotations that helped describe how participants used their homes for activity and adapted to or mitigated accessibility problems. These statements further helped establish a thorough description of the passive or proactive approaches to daily activity and subsequent adaptive environmental behaviors participants used to achieve P-E fit. Once themes and significant statements were aggregated and organized, the third author acted as an external senior analyst to further validate the credibility and trustworthiness of the findings. Parts of the findings that were found to require further discussion and action were once again revised by the first author and finally reviewed by the author team through consideration of both the qualitative and quantitative data to reach consensus.

### 2.5. Ethics

This study was conducted in accordance with the Declaration of Helsinki, and the protocol was reviewed and approved by the Institutional Review Board at Oregon State University, Corvallis, Oregon, U.S. (2013/5585). This included ethical considerations for interviews, field notes, audio recordings, observations, photographs, and the informed consent process. All participants read, signed, and were given a copy of the consent form prior to data being collected.

## 3. Findings

Successively, three themes that describe participants’ adaptive environmental behaviors to achieve objective and perceived P-E fit emerged. Behaviors were both passive and proactive to achieve comfort and optimal performance with daily necessary and desired activities. The following themes describe the adaptive approaches employed by participants to meet actual or perceived functional or environmental needs.

### 3.1. Adjusting Behavior or Attitudes to Maintain or Regulate P-E Fit

A strongly expressed or selected goal of participants was to remain in place and maximize independence in spite of functional limitations and environmental barriers that threatened accessibility and usability. The choice goal of aging in place was supported by a heightened sense of place among participants, which further served to alter behavior and attitudes toward alternative or more supportive housing options:

*This house—they’re going to carry me out in a cigar box, that’s what they’re going to do. I like where I live now. I like the furniture. I like all my junk. And everything just seems to fit okay in this house. I wouldn’t have the energy to put it in another house anyway.* (Participant 3)

*We like our home. When we got [here], I swore the only way they’d get us out of here is in a pine box. We feel good about our home and I feel good about the yard. We don’t want to leave it because this is where we live, and we like our privacy and I like having the yard to work in. So I don’t know how we’ll ever leave this place.* (Participant 6)

To maximize the possibility of remaining at home, participants actively or passively adopted particular behaviors or altered attitudes to achieve real or perceived P-E fit as a means to avoid relocation.

Passive attitudes and behaviors were exhibited among several participants unable to actively identify or recognize environmental barriers that impacted their daily function. In these cases, participants consciously adjusted their behaviors and attitudes toward accessibility and usability to optimize activity outcomes and compensate for a lack of environmental support:

*It’s not really light enough in [the living room] for me to read. Well, if I turn on all the lights and turn on that lamp, and sit in this chair, and wear those magnifying glasses, I can read in here. And of course I have a flashlight in every room and I have reading glasses in practically every room.* (Participant 3)

*You put things that you use all the time where you can get them. And you put things that you don’t use all the time up higher. For example, in my spice cabinet, the ones that I use most…they’re in alphabetical order in the first three shelves I can reach, and those are the ones most used. Now, the ones that I don’t use so often go on the top two shelves.* (Participant 12)

Although certain environmental features limited objective accessibility, participants simply reacted to these constraints by modifying the manner in which they approached, organized, facilitated, or used their spaces to accommodate daily activity. For example, instead of modifying the kitchen to accommodate all spices used for cooking, Participant 12 instead chose to deal with the cabinet the way it was and complete cooking activities within those constraints. In this way, participants described their home environments not as restrictive to accessibility, but rather requiring simple behavioral adjustments with respect to its use in order to meet daily functional and psychosocial needs.

Alternatively, proactive behavior was evident in several participants who creatively enhanced their ability to complete daily activities despite physical limitations or presence of environmental barriers:

*Well as regards the garden, there is less I can do compared to when we first moved. Now I have much more color on the deck and more plants in planters because I can’t get down to the ground and up again easily, and on that slope I feel off-balance. So I’ve gotten much more pleasure from my garden actually on the deck now, and I’ve designed it that way. Because less and less can I [garden] in ground-level beds.* (Participant 1)

*I was pretty quickly able to determine I could get to everything I needed to alone without assistance. Fortunately, I was able to scoot around on my office chair and when I got to the counter I could lift myself up to a standing position. And from there I could reach anything that I needed…you know, the microwave oven or whatever.* (Participant 11)

Selecting alternative methods or techniques to achieve daily needed and desired activities allowed some participants to overcome accessibility issues and maintain perceived usability of their home environments both inside and out. This approach, or simple adjustment of behavior without active modifications to the environment, served to manage perceived P-E fit.

Other participants described environmental barriers as manageable challenges that could proactively assist in maintaining or improving activity performance and overall health:

*Working spaces too deep…well, some of it I can’t reach too well when I’m sitting, but it’s good for me to hop up and stand.* (Participant 10)

In particular, stairs were described as positive challenges to daily functioning:

*I feel that the steps that we go through up and down stairs are good for our body. As long as we can navigate them and do them with relative ease, then we feel that they are a benefit.* (Participant 6)

*I like having the stairs; it’s good running up and down those stairs for me. That’s a challenge I can manage.* (Participant 4)

These participants were consciously aware of environmental barriers in their homes and largely agreed with objective P-E fit results. In spite of accessibility problems, however, participants maintained positive attitudes toward environmental challenges that were perceived as beneficial toward the achievement of activity performance, independence, and aging in place. In this sense, perceptions of access and use were proactively and selectively reframed based on the role of the home environment in actual or perceived health outcomes.

### 3.2. Increasing Functional or Environmental Support to Enhance P-E Fit

Several participants utilized assistive devices, products, or family members as personal or environmental supports to optimize P-E fit within unaccommodating home environments. When reflecting on objective P-E fit results, accessibility problems were discussed as easily overcome using this approach:

*Upper shelves too deep? I don’t have an issue with that with my grabber, step stool, and my husband. If those things were all missing or I was on my own, and if I got to the point where my balance got so bad I couldn’t use my step stool safely, that might be an issue.* (Participant 1)

*The cupboards [are] certainly high, but I have a step ladder next to the refrigerator and I also have a grabber stick. I like to just get the step stool out, put it in place, and get what I need. Now if I was with it, I would open the ladder, rather than just throwing it up against the side of the cabinet.* (Participant 12)

Assistive devices and assistance from family helped accommodate participant activities, thereby serving as a form of environmental support that improved usability in spite of accessibility problems. This type of strategy or behavior allowed participants to fulfill household roles, even if their choice of accommodation was not always objectively safe.

Aside from personal or product assistance, minor home modifications (e.g., task lighting, grab bars in bathrooms, railings at stairs) were sometimes added to support common activities of daily living. These provisional, short-term modifications were selected to optimize perceived ease, safety, and comfort in use for participants or their visitors:

*The showerhead in the master bathroom was too difficult for me to adjust, so we put in the removable showerhead on a bar where the height can be adjusted.We also remodeled the kitchen. We put in those round revolving shelves [lazy susans] in the pantry closet. They are so useful.* (Participant 12)

*I did have handrails put in outside and on the steps. Not so much for my benefit but for friends, my old lady friends. Yeah, they were complaining about that. And I do have some lights out there, that motion sensor, to make them safer.* (Participant 4)

Some of these modifications did not meet standards in terms of objective accessibility and were only temporary solutions to support daily activities. Regardless, they were perceived as serving participants reasonably well in terms of use and performance, thereby enhancing perceptions of P-E fit in their current state. Proactively modifying the environment in such ways was described as a means to ensure potential health or functional issues would not inhibit activity performance or prevent visitability by older friends and family.

A few participants further optimized P-E fit in their home environments by proactively selecting existing or building custom designed housing that would help support daily activities with the expressed intent or goal of aging in place. Homes were primarily chosen or designed to accommodate necessary dwelling functions on one floor. The perceived benefits of selecting a home to meet functional needs was also discussed in terms of maintaining or improving performance of daily activities and minimizing maintenance or upkeep:

*At this point I’m getting around fine, but I’m not sure that will be the case a few years ahead. But we purposely chose this single level house. We don’t have grass so I don’t have to mow grass. That was purposeful. We had a very large yard in [prior state of residence], and that was too much grass.* (Participant 9)

Other participants proactively selected independent housing on a CCRC campus that would help support functional needs over time, seeking age-friendly design features and supports that would eliminate the need for necessary modifications or assistance in the future:

*I like these light switches because I don’t have to pick and pinch at a little switch. I just rub my thumb over…change it from what it is. Showering is no problem. The shower’s designed so that I can run the wheelchair all the way in if I want to.* (Participant 10)

*I’m grateful for the fact that I don’t have to walk up steps to get in, but that’s why they’re built the way they are out here. These places are carefully thought out. They even put in a toilet that’s higher than usual. And if something goes wrong all you have to do is call maintenance and say “come and fix it!” So it’s not as if…they don’t even want you to change your light bulbs up in the ceiling.* (Participant 5)

This optimization of P-E fit through the proactive selection of a home to support functional or environmental needs was intentional, and not a consequence of passive adjustment of behaviors to meet environmental need. These participants wanted to remain independent, but understood and acted on the knowledge and awareness that additional support was needed to achieve this goal.

Comparably, several participants spoke of their selection of a home based on the opportunity for and preservation of active living and participation in the community. While the home itself was not necessarily selected for accessibility and usability, its location afforded participants a sense of security knowing that daily activities were still possible even if further limitations arose in the future:

*The very best thing about where this house is located is the fact that no matter which direction I walk, within two blocks I am where a bus will stop. I don’t have to have a car in order to survive in this neighborhood. I can walk to a store; I can ride a bus downtown or walk. I don’t have to be dependent on my driving skills to get there.* (Participant 2)

Participants expressed comfort with adequate neighborhood support (e.g., transportation services, low traffic/safe streets, and walkable community services/amenities) as a means to optimize activity performance in spite of current or potential functional or environmental limitations. In this way, selecting a home or community location based on the accommodation of potential changes in function over time helped maintain perceived usability both in and out of the home.

### 3.3. Counteracting Losses in Functional or Environmental Support to Achieve P-E Fit

Increased attachments to home and community juxtaposed with age-related decline led several participants to compensate for functional or environmental losses in order to achieve P-E fit and remain in place. This was evident in the decision of some participants to selectively implement specific and substantial modifications to their home environments in response to recent, unanticipated injury events:

*Had the handrail gone all the way to the bottom, I wouldn’t have [broken my leg and ankle from falling down the front entrance stairs] because I would have had a visual clue as to what was happening. The handrails stopped three risers short of the bottom and didn’t have the d-ring, so when I was recovering I said this has got to change. So we had a guy pull the handrail off, extend it, and put in the d-ring.* (Participant 11)

*I put some steps in down to the carport; it used to just be asphalt down there. It was a slope, and the garbage truck used to back down the driveway to pick up the garbage. And I could hear the truck rumbling and I’m running out and I took a real fall down [the asphalt slope] just as he’s going out the driveway. Really embarrassed. So I said you know, this is dangerous, this is really dangerous. So we had the steps put in which are great—something needed to be done.* (Participant 4)

In these cases, modifications were made only after the need presented itself, or when the environment could no longer support daily functioning. To counteract such loss of support, several participants subsequently modified their homes to increase accessibility and usability for the express purpose of optimizing the ability to age in place.

Compensation for limitations in function was also evident in the increased attention several participants gave toward acquiring new strategies to accessibility and usability in the home environment and/or to offset P-E fit restrictions:

*I have arthritis in my [right] shoulder. I just use my left arm. Because I can’t hardly get out of a chair sometimes and my wife pulls me with my left arm, not my right arm.* (Participant 8)

*The exterior route with steps. You don’t have to use that. Same thing coming into the house. As far as the steep exterior slopes anybody…the people [in wheelchairs] don’t go out in the backyard who have a wheelchair. They just don’t go.* (Participant 12)

In these cases, when a participant’s functional capacity did not support the performance of a specific activity, participants often compensated by capitalizing on functional abilities not impaired or impacted by age-related decline. When the environment restricted certain activities, the environmental barrier was actively avoided in favor of another feature, route, or element that compensated for accessibility and usability restrictions. Although passive and therefore yielding to functional or environmental difficulties, these types of compensatory behaviors were yet another strategy participants used to counteract functional decline and environmental challenges as a means to achieve P-E fit and remain in place.

Similarly, some participants compensated for losses in functional or environmental support by focusing on building knowledge of or planning for housing modifications or other resources needed to achieve P-E fit. These participants demonstrated awareness that their homes may need to be modified in the future when functional limitations outweighed their ability to perform needed and desired activities:

*We don’t use the bathing facilities [on the main floor]…but we may sometime. And we’ll probably have to accommodate…well I suppose a wheelchair, and that entrance is too narrow. And we’d have to step in high to get in to the bath. But like I mentioned we don’t use that bathroom. But we will sometime have to when we can’t navigate the stairs. There’s thirteen steps to get down to the bathroom.* (Participant 6)

*I’m fine [in the shower] because I have a wall for support in the bathtub. It’s not like I don’t have something I could lean on if I needed to. But [installing grab bars is] something, I myself, have thought about. So I know that that’s something we need to look into.* (Participant 2)

These compensatory behaviors were made with respect to a basic understanding of the environmental restrictions participants’ homes imposed on their daily function. This knowledge of a lack in P-E fit prescribed an increased focus on improving usability in the home through planning for future modifications. In this way, these participants exhibited adaptive compensatory behavior stemming from counteracting functional and environmental loss with an increased awareness of future P-E fit needs.

## 4. Discussion

The findings of this study shed light on the various adaptive environmental behaviors employed by functionally limited, community-dwelling U.S. older adults to achieve P-E fit. A mixed methods approach [19] using objective assessments and perceptions of P-E fit provided a thorough understanding of the P-E interaction and exemplify how older adults adapt to achieve a match between competence and environmental press. With respect to the ETA [9] and subsequent proactivity hypothesis [11], participant perceptions of accessibility and usability revealed both passive and proactive approaches to achieve performance and comfort in daily activities. Examining the objective and perceived accessibility and usability of the home environment furthermore provides insight into the adaptive environmental behaviors older adults employ to achieve P-E fit.

This study adopted a P-E fit perspective, as it is the theoretical basis for the HE instrument. Following Lawton and Nahemow’s ETA and corresponding press-competence model [9], the HE is centered on the relationship between environmental press (in the HE, environmental barriers) and personal competence (in the HE, functional limitations and dependence on mobility devices) as a means to quantify P-E fit for a resident with a particular functional profile. In this way, results from the HE instrument not only provide a rank-ordered list of environmental barriers problematic for the individual resident, but also a total P-E fit score that suggests where a resident falls within the continuum of adaptation (a phenomenon of both physical performance and personal comfort) [21]. While the person-environment-occupation (P-E-O) model [28] provides an alternative theoretical stance, it shifts the focus to meaningful occupation and the ebb and flow of P-E-O relationships over time instead of helping emphasize how older adults adapt to changes in P-E fit in their home environments through certain behaviors and strategies.

Accordingly, this study supplemented the P-E fit perspective with the *selection*, *optimization*, *and compensation* framework, or the SOC model [13] to tease out the strategies older adults use to remain independent. Reflecting upon the findings through this lens, the study shows that objectively identified accessibility problems in the home do not always stipulate or direct perceived usability. The selection and performance of daily activities depends on the means necessary or the resources perceived to accomplish these needed or desired activities. When activities are not achievable because of losses in functional or environmental support, older adults with functional limitations alter or modify objective or perceived accessibility and usability in their home environments to manage age-related decline and maintain P-E fit. This is demonstrated by the selective adjustment of behaviors or attitudes toward the performance of activities (to achieve functional goals), the utilization of assistance or environmental supports to optimize activity performance (to increase efficacy and maintain independence), and/or the implementation of modifications or alternative strategies to compensate for declines in function (to achieve resilience and regulate losses). These adaptive behaviors are both passive with respect to submitting to the constraints of the built environment and proactive in terms of using the environment as a resource to maintain or enhance function and health in older age. In this way, usability appears to be malleable with age, self-perception, and functional competency.

As such, the findings of this study align with others stating the importance of utilizing, measuring, and evaluating both objective and perceived indicators when assessing P-E fit to determine appropriate interventions or solutions that optimize independence and the ability of older adults to age in place [29]. For example, prescribing modifications to the home environments of older adults based on objective accessibility assessments alone does not provide the whole picture and may not be cost effective. Older adults perceive the usability of their home environments as related to accessibility, but simultaneously overcome objective barriers in ways that optimize and/or compensate for declines in functional or environmental support. Abiding by standardized accessibility guidelines subscribes to the passive approach, which may ultimately require flexibility in use to the extent possible based on varying functional and psychosocial needs and desires of individual residents. It is therefore important for practitioners engaged in counseling regarding environmental interventions in the home to not restrict their advice and certifications to modifications based on objective criteria but also pay close attention to resident perceptions of usability. That is, to support independence and well-being in older age, interventions should be based on a holistic P-E fit perspective.

Overall, a better understanding of the P-E interaction and the adaptive behaviors utilized by older adults in response to losses in functional or environmental support has implications for research, policy, and practice. Studying adaptive processes may serve to deepen our understanding of both P-E fit frameworks and theoretical models of aging well. The connection between adaptive behaviors employed in response to P-E fit problems and SOC processes warrants further study. Longitudinal studies exploring adaptive change over time may also help strengthen the empirical and conceptual understanding of the P-E interaction. Additionally, a better understanding of residents’ perceived accessibility, usability, and awareness of environmental barriers within their home environment can serve the development of educational programs targeting older adults and practitioners, and further guide the selection and implementation of appropriate home modifications or other interventions to achieve holistic P-E fit.

With respect to public health, understanding accessibility and the adaptive strategies older adults use to remain independent has implications for healthcare, mobility, and the ability of older adults to remain productive and engaged in daily activities both in and out of the home [30,31]. The maintenance of mobility and daily activity in older age is important for the overall health and well-being of older adults, and should be advocated for in both policy and practice. Similarly, the provision of age-friendly environments that foster healthy and active aging and alleviate fragmented and costly formal and informal care delivery to support aging in place is important [30]. Mitigating environmental barriers and increasing education surrounding positive adaptive approaches to remaining independent in older age may also serve to increase health promoting activities and engagement in positive health behaviors [32]. Public health practitioners knowledgeable in home accessibility and usability issues and corresponding integration of personal and environmental supports could contribute to the development of policies and programs aimed at assisting older adults in cooking healthier meals, maintaining a garden, visiting with family and friends, and staying active in the community, among other healthful activities.

While this study provided empirical support for the adaptive behaviors employed by older adults within their home environments, it is limited in generalizability by a number of factors. First, the sample was small, and participants were all functionally capable of living independently in ordinary or age-restricted housing (without formal support). Participants also lived in a relatively homogenous, affluent, and highly educated smaller-urban region in one specific area of the U.S. Moreover, data were collected using a cross-sectional design, which cannot capture variations of adaptive behaviors over time. Still, the findings elucidate situations that bear resemblance to the circumstances of people in similar situations, indicating the transferability of our findings.

## 5. Conclusions

This study is among the first to explore the use of objective assessments and perceptions of P-E fit in a mixed methods study to explore accessibility, usability, and the adaptive behaviors utilized by functionally limited older adults to overcome losses in functional or environmental support. Such an approach demonstrates the importance of targeting objective as well as perceived aspects of a home when exploring the interaction between people and their environments. The results could be used to develop and optimize current home modification practices. Also, the knowledge generation in this research field could benefit from applying P-E fit frameworks alongside theoretical models focusing on the adaptive capacities of older adults.
